# LncRNA LOC610012 Inhibits Canine Mammary Tumor Activity via the PTGS2/EP3 and GSK3β Signaling Pathways

**DOI:** 10.3390/cells14130974

**Published:** 2025-06-25

**Authors:** Bohan Zhang, Lixin He, Xiao Wang, Wenjing Liu, Yuxin Li, Yinan Wang, Huili Feng, Wenxuan Li, Changwei Qiu

**Affiliations:** Department of Clinical Veterinary Medicine, College of Veterinary Medicine, Huazhong Agricultural University, Wuhan 430070, China; 2022302110218@webmail.hzau.edu.cn (B.Z.); hlx2020@webmail.hzau.edu.cn (L.H.); wang-xiao@webmail.hzau.edu.cn (X.W.); lwj0916@webmail.hzau.edu.cn (W.L.); li-yuxin@webmail.hzau.edu.cn (Y.L.); wangyinan12@webmail.hzau.edu.cn (Y.W.); 2021302039008@mail.hzau.edu.cn (H.F.); liwenxuan@webmail.hzau.edu.cn (W.L.)

**Keywords:** LncRNA LOC610012, canine mammary tumors, proliferation, PTGS2

## Abstract

Canine mammary tumors (CMTs) are the common tumors in female dogs, and approximately 50% of CMTs are malignant tumors, with abnormal regulation of non-coding RNAs being a critical factor in disease progression. Currently, research on long non-coding RNAs (lncRNAs) regulating CMT development remains limited. This study identified a novel lncRNA, aiming to explore the role of lncRNA LOC610012 in CMTs. In this study, immunofluorescence and Western blot analyses were employed to detect protein expression. LncRNA LOC610012 is downregulated in CMT tissues and cells. Stable cells of LOC610012 were constructed by the lentivirus technique. Through a variety of experimental methods, LOC610012 inhibited the proliferation, invasion, and metastasis of CMT cells in in vitro and in vivo experiments conducted using cell culture and mouse models. Mechanistically, LOC610012 regulated the expression of EP3 and GSK-3β by targeting PTGS2, resulting in excessive production of reactive oxygen species (ROS), which inhibited cell viability. Similarly, through transmission electron microscopy, mitochondrial damage caused by LOC610012 was observed in CMT cells, which was manifested as mitochondrial swelling, membrane rupture, and mitochondrial ridge disappearance. PTGS2 could partially restore the inhibition of LOC610012 on cell activity. LOC610012 acts as a tumor suppressor gene in CMTs and as a potential biomarker for the disease.

## 1. Introduction

In recent years, with economic development, an increasing number of families have chosen to keep dogs to enrich their spiritual lives. Canine mammary tumors (CMTs) are one of the most common tumors in female dogs, characterized by high malignancy in existing cases and serving as a leading cause of mortality in this species [1,2]. Over 25% of intact female dogs will develop mammary tumors at some stage, with middle-aged and elderly dogs being particularly susceptible. Current treatment options for CMTs include surgical resection and adjuvant therapy [3], but adjuvant therapy is often declined by owners due to high costs [4]. However, many malignant tumors exhibit poor prognosis during treatment [5,6,7]. To improve treatment efficacy and enable early detection, extensive studies have focused on genomic mutations and transcriptomics in CMTs, yet no effective tumor biomarkers for early diagnosis are currently available.

RNA sequencing has revealed that, although the genome is extensively transcribed, only a small fraction of RNAs encode proteins. Most transcripts longer than 200 nt are classified as long non-coding RNAs (lncRNAs) [8]. LncRNAs are capable of regulating transcription, epigenetic modifications, chromatin structure, and translation through interactions with DNA, RNA, proteins, and even signaling receptors [9]. Emerging evidence has demonstrated the involvement of lncRNAs in canine mammary tumors, canine B-cell lymphoma, and canine melanoma [10,11,12,13,14].

PTGS2, also known as cyclooxygenase-2 (COX-2), was discovered in 1991 and functions as a membrane-bound enzyme and rate-limiting enzyme [15]. PTGS2 is typically undetectable in healthy tissues and organs. In adults, it is only present in the central nervous system, kidneys, vesicles, and placenta, while in fetuses, it is found in the heart, kidneys, lungs, and skin. PTGS2 is a highly inducible isoenzyme that can be rapidly upregulated by various pro-inflammatory factors, including cytokines, tumor promoters, and mitogens, particularly in cells involved in inflammation, pain, fever, tumors, Alzheimer’s disease, or osteoarthritis. As such, it has long been considered an important target for pain relief and inflammation treatment [16]. Additionally, PTGS2, as prostaglandin–endoperoxide synthase 2, is responsible for generating prostaglandins such as prostaglandin E2 (PGE2), which play regulatory roles in various cancers. For instance, PTGS2 is upregulated in adenocarcinoma of the gall bladder, squamous cell carcinoma, cholangiocarcinoma, transitional cell carcinoma, endometrial carcinoma, and hepatocellular carcinoma [17]. The tumor microenvironment is a key inducer of PTGS2 overexpression, which arises from imbalances in transcriptional or post-transcriptional regulation, making it a promising biomarker for tumor identification [18]. Studies have reported a novel oncogenic mechanism of lncRNA HULC, driving abnormal tumor proliferation through the “HULC-USP22-PTGS2” molecular regulatory circuit in hepatocellular carcinoma (HCC) [19]. Mechanistically, HULC enhances the stability of the deubiquitinase USP22, selectively removing K48-linked polyubiquitination from the PTGS2 protein and blocking the proteasomal degradation pathway, thereby extending the half-life of the PTGS2 protein. This finding successfully explains the clinical paradox of elevated PTGS2 protein levels without corresponding mRNA changes in HCC.

Glycogen synthase kinase-3 (GSK-3) is an evolutionarily conserved serine/threonine kinase first identified in mammals [20]. Studies have shown that GSK-3 phosphorylates nearly 100 substrates and influences numerous cellular processes, including proliferation and the regulation of metabolic and signaling pathways. GSK-3 was initially isolated from rabbit skeletal muscle extracts, and two highly homologous isoforms, GSK-3α and GSK-3β, were identified in mammals [21]. These isoforms differ in their N-terminal domains but share 98% sequence homology in their kinase core domains, with GSK-3β exhibiting high expression in the central nervous system [22]. Knockout studies revealed that mice lacking GSK-3α can survive, while GSK-3β deficiency leads to embryonic lethality, indicating that GSK-3β has non-redundant biological functions during development [23]. It has been demonstrated that PTGS2 stabilizes the p120-catenin/β-catenin complex by inhibiting GSK-3β phosphorylation, maintaining E-cadherin membrane localization through a non-canonical pathway, and promoting cluster aggregation of circulating tumor cells (CTCs), thereby enhancing their metastatic colonization capacity in an inflammatory microenvironment [24].

Reactive oxygen species (ROS) in tumor cells are primarily generated by NADPH oxidases of the NOX family and mitochondrial respiratory chain complexes (MRCCs). Previous research has suggested that ROS may serve as potential targets for cancer therapy. The effects of ROS on cancer cells are highly complex, depending on the level of ROS stress and the metabolic and genetic context of the cancer cells. Recent studies indicate that due to the generally elevated ROS levels in cancer cells, ROS-mediated post-translational modifications and epigenetic changes can lead to alterations in transcription, signal transduction, and metabolism. Therefore, ROS-based cancer therapy is based on redox signaling, cancer metabolism, and its impact on cancer immunity [25]. Jin et al. (2019) found that high expression of pSer9-GSK-3β induces higher ROS levels and observed that abnormal function of mitochondrial respiratory chain complexes I/III induced by GSK-3β disrupts electron transport chain function, thereby perturbing ROS homeostasis [26].

LncRNA LOC610012 is an lncRNA discovered by our research group in previous studies. Through in vivo and in vitro experiments, this study found that LOC610012 induces the downregulation of EP3 and GSK3β expression by inhibiting the expression of PTGS2, further leading to the excessive accumulation of intracellular ROS, ultimately inhibiting the activity of canine breast cancer cells. This study identified LOC610012 as a key regulatory molecule in the regulation of CMT proliferation and invasion, providing a new theoretical basis for the molecular targeted therapy of CMTs.

## 2. Materials and Methods

### 2.1. Antibodies

The antibodies used were as follows: β-actin (ABclonal, Wuhan, China), NF-κBp65 and p-IκB (Abmart, Wuhan, China), p-NF-κBp65 (Wanleibio, Shenyang, China), GSK-3β and p-GSK-3β (WANLEIBIO, Shenyang, China), EP3 (ABbox, Suzhou, China), and PTGS2 (HUABIO, Hangzhou, China), HRP-conjugated Goat anti-Rabbit IgG (H + L) (Abclonal, Wuhan, China), and Alexa Flour 594-Goat Anti-Rabbit IgG (Servicebio, Wuhan, China).

### 2.2. Clinical Sample Collection and Cell Culture

Three pairs of fresh CMT tissues and adjacent normal mammary tissues were collected clinically, along with associated pathological data including age, breed, tumor size, and calcification status. The cell lines CHMp, CMT7364, and 293T were cultured in DMEM (Gibco, Billings, MA, USA) supplemented with 10% FBS (Hycezmbio, Wuhan, China) and 100 U/mL penicillin–streptomycin (Gibco, Billings, MA, USA) at 37 °C in a 5% CO_2_ atmosphere.

### 2.3. RNA Extraction and RT-qPCR

Tissue RNA was extracted using TRIZOL Reagent (Vazyme Biotech, Nanjing, China) with a tissue grinder (Shanghai Jingxin Industrial Development, Shanghai, China). Cell RNA was extracted directly using TRIZOL Reagent. Reverse transcription was performed using a reverse transcription kit (ABclonal, Wuhan, China), and qPCR was conducted using Taq Pro Universal SYBR qPCR Master Mix (Vazyme Biotech, Nanjing, China) with GAPDH as an internal reference.

Primer sequences used in this study are listed in Table 1.

### 2.4. Overexpression of LncRNA LOC610012 in Cells

Lentiviruses encapsulating the overexpression plasmids pCD513B-LOC610012 (oe-LOC610012) and pCD513B-NC (oe-NC) (Gene Create, Wuhan, China) were constructed by transfecting 293T cells with jetPRIME (Polyplus, Illkirch, France) along with pLP1, pLP2, and pLP-VSVG plasmids. CHMp and CMT7364 cells were cultured in medium containing 293T-conditioned medium for 24 h, followed by culture in 2% FBS medium for an additional 24 h. Cells were then trypsinized and seeded into 12-well plates, and stable cell lines overexpressing LOC610012 (oe-LOC610012) and control lines (oe-NC) of CHMp and CMT7364 were established using complete medium containing 5 μg/mL puromycin.

### 2.5. Overexpression and Knockdown of PTGS2

The overexpression plasmid pcDNA3.1-PTGS2 and small interference RNAs (siRNAs) targeting PTGS2 (Gene Pharma, Shanghai, China) were transfected into CHMp and CMT7364 cells using jetPRIME. Transfection efficiency was evaluated 24 h post-transfection.

siRNA sequences for PTGS2 (si-PTGS2) and the negative control (si-NC) are provided in Table 2.

### 2.6. Cell Viability Assay

Cells were seeded into 96-well plates at a density of 5 × 10^3^ cells per well. Cell viability was assessed at 0, 12, 24, 36, and 48 h using the Cell Counting Kit-8 (CCK-8, Hycezmbio, Wuhan, China). Absorbance at 450 nm was measured using a multifunctional microplate reader (BMG LABTECH, SPECTORstar, Offenburg, Germany).

### 2.7. Wound-Healing Assay

To quantify cell migration, cells were cultured to 80% confluence in 6-well plates. A scratch was created using a 200 μL pipette tip, and non-adherent cells were removed by washing twice with sterile PBS. Cells were then cultured in DMEM with 2% FBS. Images of the scratch were captured immediately and after 24 h using an inverted microscope (Soptop, ICX4I, Ningbo, China), and the distance of the scratch was measured using ImageJ 1.53e software.

### 2.8. Transwell Assay

For invasion assays, Matrigel (Biozellen, Ord, NE, USA) was diluted to 1/20 of the original volume and coated onto the upper chambers of 24-well transwell inserts (Labselect, Hefei, China). After Matrigel solidification at 4 °C, cells were trypsinized, resuspended in 100 μL of low-serum medium, and seeded into the upper chambers at densities of 1 × 10^4^ (CHMp) and 3 × 10^4^ (CMT7364) cells per well. Complete medium (500 μL) was added to the lower chambers. After 24 h of incubation, invasive cells on the underside of the upper chambers were fixed with methanol, stained with crystal violet, and imaged using an optical microscope (Olympus, Tokyo, Japan).

### 2.9. In Vivo Tumorigenicity Assay

This study was approved by the Institutional Animal Care and Use Committee of Huazhong Agricultural University (HZAUMO-2025-0033) and conducted in accordance with NIH guidelines. Four-week-old female Balb/C mice were purchased from the Experimental Animal Center of Huazhong Agricultural University (Wuhan, China) and acclimated for one week under standard conditions. Approximately 1 × 10^6^ CHMp cells (oe-NC group and oe-LOC610012 group) were collected, resuspended in 100 μL PBS, and subcutaneously injected into the dorsal region of mice. After two weeks, all mice were euthanized, and xenograft tumors were excised, measured for diameter and weight, with portions fixed in 4% paraformaldehyde and stored at −80 °C.

### 2.10. Colony Formation Assay

Stably transfected CHMp and CMT7364 cells were seeded into 6-well plates at a density of 1 × 10^3^ cells per well and cultured for 14 d with medium changes every three days. After fixing with 4% paraformaldehyde and staining with crystal violet, colonies were imaged.

### 2.11. Intracellular ROS Detection

Cells cultured to 90% confluence in 6-well plates were incubated with a 1/2000 dilution of the ROS detection reagent (BestBio, Shanghai, China) at 37 °C for 30 min in the dark. After washing twice with PBS, images were captured using an inverted fluorescence microscope (Olympus, Tokyo, Japan).

### 2.12. RNA-Seq Analysis of Downstream Genes

To investigate the molecular mechanisms of LOC610012, RNA sequencing (RNA-Seq) was performed on oe-NC and oe-LOC610012 cells of CHMp (Sequence Read Archive (SRA) submission: SUB15160414). Triplicate samples of each group were cultured to 90% confluence, washed three times with PBS, trypsinized, and collected into 1.5 mL tubes. Samples were sent to Novogene (Beijing, China) for sequencing on the MiSeq 2000 platform. Differentially expressed genes were identified using a threshold of padj ≤ 0.05 and |log_2_FoldChange| ≥ 1.0, and visualized using volcano plots and heatmaps. Functional enrichment analysis was performed using KEGG and GO databases and visualized with scatter plots.

### 2.13. Immunofluorescence Staining

Cells were seeded into 24-well plates at a density of 1 × 10^4^ cells per well. After reaching 80% confluence, cells were fixed with 4% paraformaldehyde for 20 min, permeabilized with 0.2% Triton X-100 for 2 h, and blocked with 5% BSA for 2 h. Primary antibodies were applied overnight at 4 °C, followed by incubation with Alexa Flour 594-Goat Anti-Rabbit IgG for 2 h in the dark. Nuclei were stained with DAPI (Beyotime, Shanghai, China) for 15 min, and images were captured using an inverted fluorescence microscope (Olympus, Tokyo, Japan).

### 2.14. Western Blot Analysis

Cells were lysed in RIPA buffer (Biosharp, Hefei, China) containing 1% phosphatase inhibitors (Applygen, Beijing, China) and 1% protease inhibitors (PMSF, Biosharp, Hefei, China). Total protein concentration was measured using the bicinchoninic acid (BCA) protein assay kit (Hycezmbio, Wuhan, China). Samples were normalized to 20 μg of protein, separated by PAGE gel electrophoresis using a PAGE Gel Quick Preparation Kit (Yeasen, Shanghai, China), transferred to PVDF membranes, and blocked with 5% skim milk for 2 h. Images were acquired using a Fusion Solo S imaging system (Viber, Paris, France).

### 2.15. Electron Microscopy for Mitochondrial Morphology

Cells cultured to 80% confluence in flasks were fixed with electron microscopy fixative for 5 min in the dark, detached using a cell scraper, centrifuged, and resuspended in fresh fixative. Samples were processed successively: fixed, dehydrated, and permeated. Resin blocks containing samples were cut into ultrathin sections (80 nm thick) with a Leica UC6 ultrathin microtome. Ultrathin sections were observed using a transmission electron microscope (TEM) (H7650, HITACHI, Tokyo, Japan) at 100 kV after being stained with uranium acetate.

### 2.16. Statistical Analysis

Data are presented as the mean ± standard deviation (SD) from at least three independent experiments. Statistical comparisons were performed using one-way ANOVA and *t*-tests with GraphPad Prism 9. A *p*-value < 0.05 (*) was considered statistically significant, <0.01 (**) highly significant, and >0.05 non-significant.

## 3. Results

### 3.1. LOC610012 Is Downregulated in CMT Tissues and Cells

**Figure 1:**
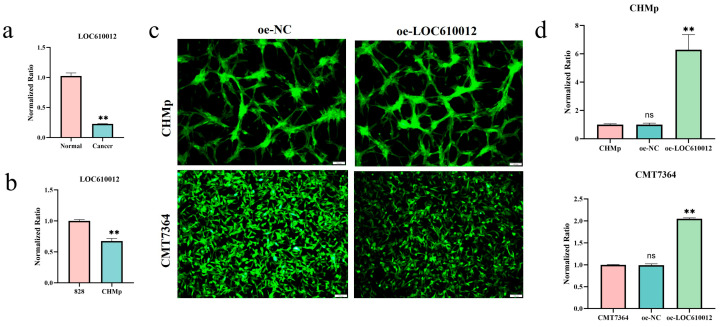
LOC610012 is downregulated in CMT tissues and cells. (**a**) RT-qPCR was used to detect LOC610012 expression between canine mammary and CMT tissues. (**b**) Changes of LOC610012 expression between primary cells of normal canine mammary gland and CMT cells. (**c**) The intracellular GFP signal was observed by fluorescence microscopy in stably transfected cell lines of CHMp and CMT7364; scale bar: 100 μm. (**d**) RT-qPCR was used to detect the expression of LOC610012 in stably transfected cell lines of CHMp and CMT7364. All results are expressed as mean ± SD of three independent experiments. ** *p* < 0.01, ns = not significant.

Clinically, three cases of primary canine mammary tumors were collected. After extracting tissue RNA, the difference in the expression of LOC610012 between normal mammary tissues and tumor tissues was detected by RT-qPCR. Canine normal mammary cells named 828 were isolated and cultured, and their RNA was extracted. Meanwhile, the RNA of CHMp was also extracted. The difference in the expression of LOC610012 in the cells was detected by RT-qPCR. The results all showed that LOC610012 was in a low-expression state in tumor tissues and cells (Figure 1a,b). Stable cell lines overexpressing LOC610012 were established via lentiviral transduction (Figure 1c), confirmed by RT-qPCR (Figure 1d).

### 3.2. LOC610012 Inhibits CMT Cell Proliferation, Migration, Invasion, and Tumorigenicity In Vitro and In Vivo

**Figure 2:**
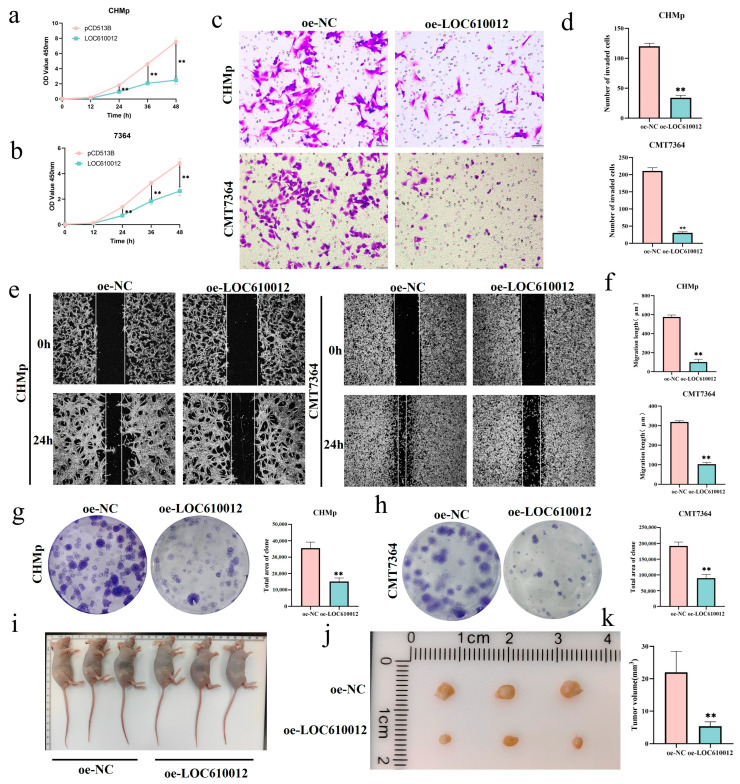
LOC610012 inhibits CMT cell proliferation, migration, invasion, and tumorigenicity in vitro and in vivo. (**a**,**b**) CCK-8 was used to detect the effect of LOC610012 on cell viability at 0, 2, 24, 36, and 48 h. (**c**,**d**) Cell invasion capacity was detected by invasion chamber assay; scale bar: 50 μm. (**e**,**f**) Relative mobility was detected using a scratch assay after LOC610012 overexpression; scale bar: 200 μm. (**g**,**h**) The clone formation assay was used to detect the rate of cell cloning. (**i**–**k**) After 14 d, the tumor volume of the oe-NC group and oe-LOC610012 group in CHMp changed. All results are expressed as mean ± SD of three independent experiments. ** *p* < 0.01.

To investigate the regulatory role of LOC610012 in tumors, a series of in vitro experiments were conducted. The CCK-8 assay was used to detect the cell proliferation rate. The results showed that the overexpression of LOC610012 significantly inhibited the proliferation of CMT cells compared with the control group (Figure 2a,b). The wound-healing assay and the transwell assay indicated that the overexpression of LOC610012 inhibited the migration and invasion ability of tumor cells, respectively (Figure 2c–f). The colony formation assay results showed that, compared with the control group, the overexpression of LOC610012 significantly decreased the colony formation rate of tumor cells (Figure 2g,h). Collectively, LOC610012 significantly inhibited the viability of canine mammary tumor cells.

A subcutaneous tumorigenesis model in nude mice was successfully established by subcutaneously injecting oe-NC and oe-LOC610012 cells of CHMp. The tumor volume was measured every two days. The data showed that the size and weight of tumors in the oe-LOC610012 group were significantly lower than those in the oe-NC group (Figure 2i–k). In conclusion, the overexpression of LOC610012 inhibited the proliferation and tumorigenic ability of mammary cells in vivo.

### 3.3. RNA-Seq Identifies PTGS2 as a Key Downstream Target of LOC610012

**Figure 3:**
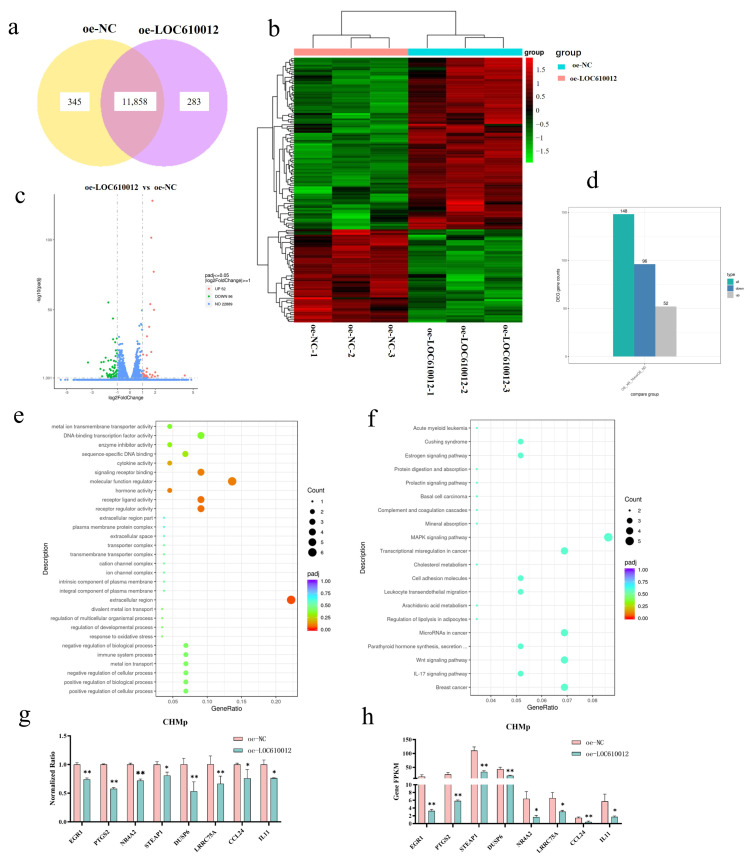
RNA-Seq identifies PTGS2 as a key downstream target of LOC610012. (**a**) Venn diagram shows the number of genes shared by oe-NC and oe-LOC610012 in CHMp. (**b**) Cluster heatmap displaying expression patterns of DEGs. (**c**,**d**) Volcano plot shows the distribution of upregulated and downregulated DEGs. (**e**) GO enrichment results of DEGs. (**f**) KEGG enrichment results of DEGs. (**g**) Changes in expression of eight genes selected from the sequencing results. (**h**) The FPKM values of the eight genes selected from the test samples were changed, and FPKM is usually used to compare the expression of the same gene under different conditions. All results are expressed as mean ± SD of three independent experiments. * *p* < 0.05, ** *p* < 0.01.

To investigate the underlying mechanisms by which LOC regulates the proliferation of CMT cells, RNA-Seq was performed to profile the transcriptomic changes induced by LOC610012. RNA-seq results indicated that there were certain differences between the two submitted groups of samples. A total of 12,486 genes were detected through sequencing (Figure 3a,b). Compared with the control group, the overexpression of LOC610012 led to the differential expression of 148 genes (padj ≤ 0.05, |log2FoldChange| ≥ 1.0). Among them, 96 genes were downregulated and 52 genes were upregulated (Figure 3c,d). GO enrichment analysis and KEGG enrichment analysis were performed on the differentially expressed genes obtained from sequencing. The results showed that LOC610012 mainly functioned in the pathways regulating receptor–ligand activity and biological processes (Figure 3e,f). Eight genes were selected from the sequencing results for validation. RT-qPCR results showed that the PTGS2 mRNA level in the oe-LOC610012 group was significantly lower than that in the oe-NC group, and this trend was consistent in both CHMp and CMT7364 (Figure 3g,h). The overexpression of LOC610012 led to a decrease in PTGS2 mRNA expression, suggesting that LOC610012 may inhibit the progression of CMT by suppressing the expression of PTGS2.

### 3.4. LOC610012 Downregulates PTGS2 to Inhibit EP3 and Promote GSK-3β Phosphorylation

**Figure 4:**
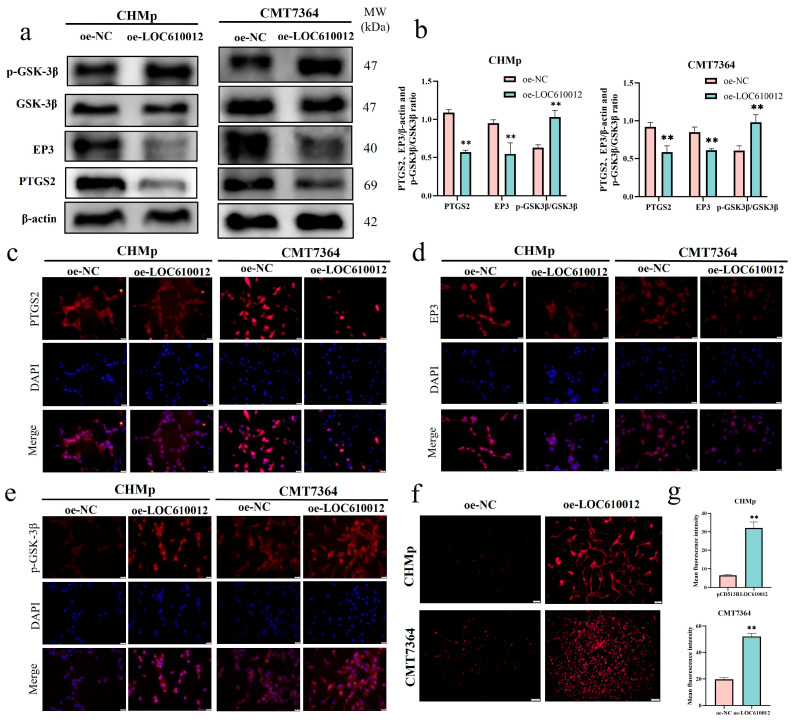
LOC610012 downregulated PTGS2 to inhibit EP3 and promote GSK-3β phosphorylation. (**a**,**b**) PTGS2, EP3, GSK-3β, and p-GSK-3β protein expression was detected by Western blotting after LOC610012 overexpression. (**c**) The expression level of PTGS2 after LOC610012 overexpression was detected by immunofluorescence; scale bar: 20 μm. (**d**) The expression level of EP3 after LOC610012 overexpression was detected by immunofluorescence; scale bar: 20 μm; (**e**) The expression level of p-GSK-3β after LOC610012 overexpression was detected by immunofluorescence; scale bar: 20 μm. (**f**,**g**) The changes in ROS levels were observed by fluorescence microscopy; scale bar: 100 μm. All results are expressed as mean ± SD of three independent experiments. ** *p* < 0.01.

The RNA-seq results showed that among the downstream genes of PTGS2, EP3 (PTGER3) had a significant difference. Studies have shown that PTGS2 can regulate the expression level of GSK-3β [24]. This implies that LOC610012 inhibits the activity of CMT cells through the PTGS2/EP3&GSK-3β axis. To verify this hypothesis, Western blotting and immunofluorescence assays were conducted. The experimental results showed that compared with the control group, the expression levels of PTGS2 and EP3 in the oe-LOC610012 group decreased, and correspondingly, the protein expression level of GSK-3β increased significantly in CHMP and CMT7364 cells (Figure 4a–e). This indicated that LOC610012 inhibits the activity of canine mammary tumor cells through the PTGS2/EP3&GSK-3β axis.

### 3.5. LOC610012 Induces ROS Accumulation

Excessive accumulation of ROS can lead to cell damage, so the ROS level is relatively low in normal cells. Studies have shown that the oxidative stress state can be regulated by modulating the expression of GSK-3β [27]. The overexpression of LOC610012 promotes the expression of GSK-3β by reducing the expression of PTGS2, which indicates that the intracellular ROS content may change. To verify this hypothesis, we measured the ROS levels in the oe-NC group and the oe-LOC610012 group in CHMp and CMT7364 cells. The experimental results showed that the intracellular ROS level in the oe-LOC610012 group was higher than that in the control group (Figure 4f,g), which verified the previous hypothesis.

### 3.6. PTGS2 Overexpression Partially Rescues LOC610012-Mediated ROS Accumulation

**Figure 5:**
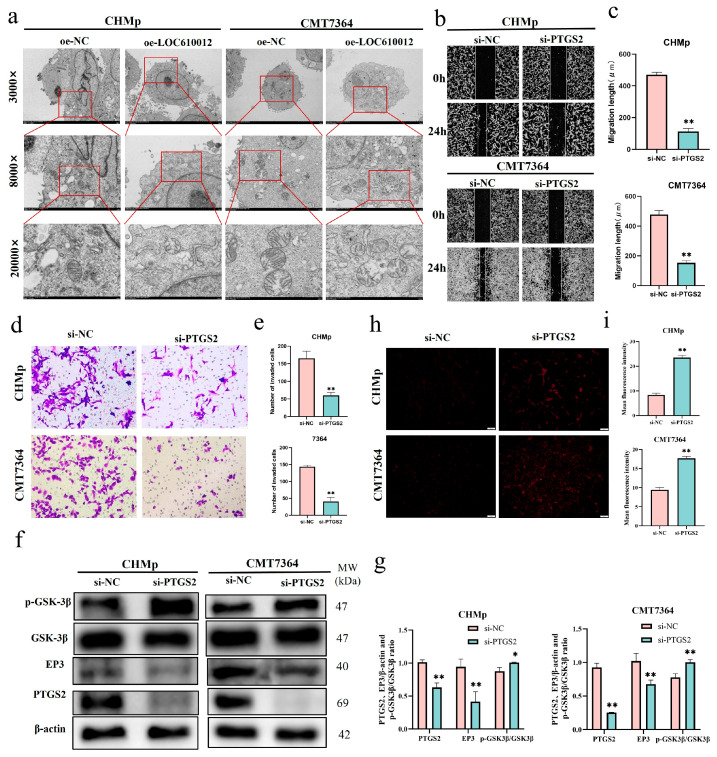
PTGS2 knockdown reduced cell viability in CHMp and CMT7364. (**a**) Mitochondrial morphology was observed by transmission microscopy; zoom multiple and scale bar: 3000×, 5 μm; 8000×, 2 μm; 20,000×, 500 nm. (**b**,**c**) The scratch test was used to detect the relative mobility after PTGS2 knockdown; scale bar: 200 μm. (**d**,**e**) After PTGS2 knockdown, cell invasion ability was detected by an invasion chamber assay; scale bar: 50 μm. (**f**,**g**) Western blotting was used to detect the phosphorylation levels of EP3 and GSK-3β after PTGS2 knockdown. (**h**,**i**) After PTGS2 knockdown, the intracellular ROS level was detected by fluorescence microscopy; scale bar: 100 μm. All results are expressed as mean ± SD of three independent experiments. * *p* < 0.05, ** *p* < 0.01.

An increase in the intracellular ROS level often indicates mitochondrial damage. We hypothesized that the mitochondria in the oe-LOC610012 group of CHMp and CMT7264 cells would be in an abnormal state. The experimental results showed that, compared with the control group, in the oe-LOC610012 group, there was partial mitochondrial dissolution, cristae rupture, and uneven distribution of the mitochondrial matrix (Figure 5a). The experimental results verified the previous hypothesis of mitochondrial damage.

To verify that the overexpression of LOC610012 reduces the expression of PTGS2, further leading to changes in the activity of CMT cells, this study designed a rescue experiment. First, the function of PTGS2 in CMT was investigated. SiRNA targeting PTGS2 was transfected into CHMp and CMT7364 cells, and wound-healing and transwell assays were conducted. The experimental results showed that interfering with the expression of PTGS2 could significantly inhibit the migration and invasion abilities of the cells, which was consistent with the results of the LOC610012 overexpression group (Figure 5b–e). Meanwhile, the Western blotting results showed that when the protein expression of PTGS2 decreased, the protein expression of EP3 also decreased, and the protein expression of p-GSK-3β increased significantly (Figure 5f,g). By detecting the ROS level using a fluorescent probe, it was found that compared with the si-NC group, the intracellular ROS level in the si-PTGS2 group increased significantly (Figure 5h,i). This indicates that PTGS2 acted on CHMp and 7364 cells through the EP3 and p-GSK-3β.

**Figure 6:**
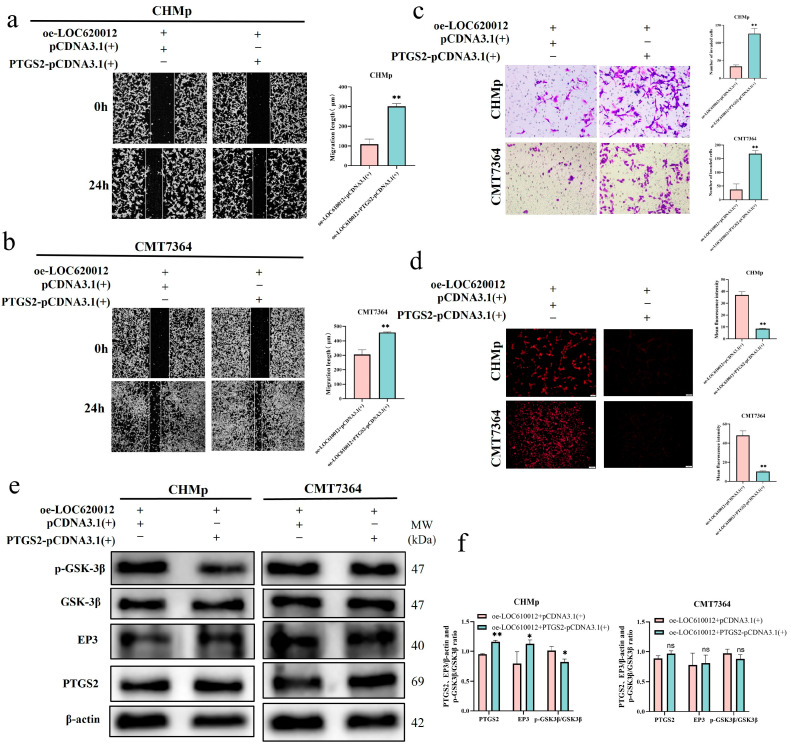
PTGS2 overexpression partially rescues LOC610012-mediated decline in cell viability. (**a**,**b**) The relative mobility of CHMp oe-LOC610012 and 7364 oe-LOC610012 after PTGS2 overexpression was measured using a scratch assay; scale bar: 200 μm. (**c**) The relative mobility of CHMp oe-LOC610012 and 7364 oe-LOC610012 after PTGS2 overexpression was detected by an invasion chamber assay; scale bar: 50 μm. (**d**) Fluorescence microscopy was used to detect the changes in intracellular ROS levels after PTGS2 was overexpressed by CHMp oe-LOC610012 and 7364 oe-LOC610012; scale bar: 100 μm. (**e**,**f**) Western blotting was used to detect the phosphorylation levels of EP3 and GSK-3β after PTGS2 overexpression in CHMp oe-LOC610012 cells. All results are expressed as mean ± SD of three independent experiments. * *p* < 0.05, ** *p* < 0.01, ns = not significant.

To determine whether LOC610012 exerted its tumor-suppressive effect through PTGS2, we performed a PTGS2 rescue experiment. We transfected PTGS2-pCDNA3.1 (+) into the oe-LOC610012 group in CHMp and CMT7364 cells to observe whether the overexpression of PTGS2 could rescue the inhibitory effect of LOC610012 overexpression on cell activity. Wound-healing assays, transwell assays, and intracellular ROS level detection experiments were conducted. The results showed that compared with the oe-LOC610012 + NC-pCDNA3.1 (+) group, the overexpression of PTGS2 in oe-LOC610012 cells partially restored the migration and invasion abilities of tumor cells and significantly reduced the increase in ROS induced by LOC610012 overexpression (Figure 6a–d). This indicated that the rescue of PTGS2 could partially alleviate the inhibitory effect of LOC610012 overexpression on cell activity. Western blotting results showed that compared with the oe-LOC610012 + NC-pCDNA3.1 (+) group, the expression of EP3 in the oe-LOC610012 + PTGS2-pCDNA3.1 (+) group showed an upward trend, while the phosphorylation level of GSK-3β showed a downward trend (Figure 6e,f). It indicates that the overexpression of PTGS2 can counteract the effects of LOC610012 overexpression on EP3 and GSK-3β.

## 4. Discussion

LncRNAs are RNA molecules longer than 200 nucleotides without protein-coding potential [8]. Emerging evidence highlights their critical roles in tumorigenesis and progression, with lncRNA-mediated metabolic networks representing one of the fastest cellular responses to internal and external stimuli [28]. LncRNAs participate in diverse biological processes, including protein interactions, epigenetic regulation, and translational control, exerting either oncogenic or tumor-suppressive effects in cancer [29,30,31].

Our previous study identified one lncRNA LOC610012 with regulatory potential in breast tumors. In order to verify whether LOC610012 has a regulatory role in canine mammary tumors, we detected that the expression of LOC610012 was significantly decreased in canine mammary tumor cells and normal mammary epithelial tissues, indicating that OLOC610012 has a regulatory role in the occurrence and development of canine mammary tumors. It can be used as a biological indicator of cancer.

Studies have shown that abnormally expressed lncRNAs in tumor tissues have different regulatory effects on tumor cell proliferation, apoptosis, invasion, and metastasis [32]. For example, LINC01133 inhibits the proliferation, invasion, and metastasis of gastric cancer cells by directly targeting miR-106-3p and then by inactivating the IPC/Wnt/β-catenin pathway [33]. In order to verify the role of LOC610012 in canine breast tumor cells, LOC610012 overexpression vectors were stably transferred to CHMp and CMT7364, and cell function tests were performed. The results showed that LOC610012 could inhibit the proliferation, migration, invasion, and tumorigenesis of CMT cells in vitro, and inhibit the proliferation of canine mammary tumor cells in vivo. The role of LOC610012 as a tumor suppressor gene in canine breast tumors was verified.

In order to explore the mechanism of LOC610012 inhibiting the proliferation and migration of CMT, high-throughput RNA-seq and GO, KEGG pathway enrichment analysis were conducted. The results showed that LOC610012 is mainly involved in regulating receptor ligand activity and biological processes. This provided important clues for us to select the downstream gene of LOC610012. Through screening, we found that PTGS2 was most affected by LOC610012. Based on this finding, we further investigated whether PTGS2 is the target gene of LOC610012 in canine breast tumors, and whether LOC610012 exerts its tumor suppressor effect by regulating PTGS2.

In 1991, a coinducible COX named COX-2 was isolated [34], also known as PTGS2. The human PTGS2 gene is located on chromosome 1 (1q25.2-25.3) and is about 8.3 kb long [35]. PTGS2 is expressed in many types of cancer and plays a multifaceted role in carcinogenesis or promotion of carcinogenesis and the resistance of cancer cells to chemical and radiation therapies. PTGS2 induces the activity of cancer stem cells (CSCs) and promotes apoptotic resistance, proliferation, angiogenesis, inflammation, invasion, and metastasis of cancer cells, suggesting that inhibition of PTGS2 could play a role in the treatment of cancer [36]. Studies have shown that inhibiting the expression of PTGS2 can inhibit the proliferation, migration, and invasion of breast tumors [37], which is consistent with our experimental results above.

Bioinformatics analysis and experimental validation showed that PTGS2 downregulation by LOC610012 leads to reduced EP3 expression and increased GSK-3β phosphorylation. EP3, a PGE2 receptor, has been linked to tumor cell proliferation and survival [38]. GSK-3β, a serine/threonine kinase, plays dual roles in cancer, with its activation often associated with tumor suppression [39,40]. Our results align with Balamurugan et al. (2023) [24], who demonstrated PTGS2-mediated regulation of GSK-3β in metastatic breast cancer.

GSK-3β and GSK-3α are evolutionarily conserved serine/threonine kinases with distinct N-terminal domains but sharing 98% homology in their internal kinase domains, originally identified in mammals [39]. Studies have shown that GSK-3β is directly involved in cell death induced by PI3K/mTOR inhibitors and panhistone deacetylase inhibitors in lymphoma cell lines [40]. Furthermore, GSK-3β can influence cancer cell apoptosis, as demonstrated in MCF7 breast cancer cells where trichostatin A, a histone deacetylase inhibitor (HDACI), induces apoptosis through GSK-3β activation [41]. Notably, GSK-3β expression levels are regulated by the PTGS2 gene [24], which aligns with our experimental findings. Through experiments, we verified that LOC610012 inhibits PTGS2 to suppress EP3 and promote GSK-3β expression.

GSK-3β is of interest because of its regulatory role in mitochondrial function and oxidative stress, and inhibition of GSK-3β improves mitochondrial function and inhibits oxidative stress [42]. This suggests that GSK-3β may influence cell activity by regulating mitochondrial function and oxidative stress in canine breast tumor cells. The experimental results also confirmed our hypothesis above. When GSK-3β is upregulated, intracellular ROS levels increase accordingly, indicating dysregulation of intracellular oxidative stress. And an increase in intracellular ROS levels is often accompanied by mitochondrial damage. According to the observation of electron microscope sections, the mitochondria in the LOC610012 overexpression group were damaged to different degrees compared with the control group. These experiments indicated that LOC610012 affected mitochondrial function and morphology through the PTGS2/GSK-3β axis. When we knocked out PTGS2 in CHMp and CMT7364, cell activity was inhibited and EP3 expression decreased while GSK-3β expression increased. However, when we overexpressed PTGS2 in CHMp-LOC610012 and CMT7364-LOC610012, the cell activity and the expression levels of EP3 and GSK-3β returned to a relatively normal state, which verified the results obtained in the above experiments.

Rescue experiments further validated the PTGS2/EP3&GSK-3β axis, with PTGS2 overexpression reversing LOC610012-mediated effects on cell migration, invasion, and ROS levels. These results confirm that LOC610012 exerts its tumor-suppressive effects through PTGS2 regulation.

Currently, various human-related lncRNAs are regarded as tumor prognostic markers. For example, H19 can be used as a prognostic indicator for human breast cancer patients [43], but no such tumor diagnostic or prognostic markers have been identified in canines. This study elucidated the underlying mechanism by which LOC610012 regulated the activity of CMT cells through PTGS2. Based on this mechanism, the next research direction could involve collecting veterinary clinical cases and detecting the expression level of LOC610012 in CMT tissues, analyzing the clinical relevance between LOC610012 and the diagnostic accuracy and prognosis of CMTs, so as to provide important support for LOC610012 as a diagnostic and prognostic marker for CMTs.

## 5. Conclusions

This study is the first to demonstrate that lncRNA LOC610012 inhibits CMT cell proliferation, migration, and invasion, as well as tumor growth in vivo, by regulating the PTGS2/EP3/GSK-3β axis. These findings highlight LOC610012 as a potential biomarker and therapeutic target for CMTs. Future research will focus on further elucidating the precise mechanisms underlying PTGS2 regulation in CMTs.

## Figures and Tables

**Table 1:** Primer sequences for RT-qPCR.

Primers Name	Sequence (5′-3′)
LncRNA LOC610012 (forward)	TGGCAGCGTCTGTAACTGAA
LncRNA LOC610012 (reverse)	AGGGCAACTTTACTGGGAACA
PTGS2 (forward)	GCGGGAGCATAACAGAGTGT
PTGS2 (reverse)	GCTCGTCTGGAATAACCGCT
EGR1 (forward)	GCCACCACATACTCTTCCGT
EGR1 (reverse)	TTGTCATGTCCGAAAGCCCT
NR4A2 (forward)	GTATGGGTCCTCGCCTCAAG
NR4A2 (reverse)	AGCCTGTGCTGTAGTTGTCC
STEAP1 (forward)	AGGAGACACCAGAGTGCTGA
STEAP1 (reverse)	AAGCTCGGCAGGACAATCAA
DUSP6 (forward)	GTTGGAGGAGTTCGGCATCA
DUSP6 (reverse)	GACACCACAGTTTTTGCCCC
LRRC75A (forward)	CAAGCGATCCCCAAAACAGG
LRRC75A (reverse)	GTCTGAACCGCACCTACAGT
CCL24 (forward)	CTGCACTGGGTCCAGAAGTT
CCL24 (reverse)	AAGCTTGGGGCATCTCTGC
IL11 (forward)	AGCCGCTCTCTTCTTGTGTC
IL11 (reverse)	TTCCCTCGTGTAAGGCACAG
GAPDH (forward)	TGACACCCACTCTTCCACCTTC
GAPDH (reverse)	CGGTTGCTGTAGCCAAATTCA

**Table 2:** siRNA sequences.

siRNA Name	Sequence (5′-3′)	
si-PTGS2	Sense	GCUUUAGGCUGAAGCCCUATT
	Antisense	UAGGGCUUCAGCCUAAAGCTT
si-NC	Sense	UUCUUCGAACGUGUCACGUTT
	Antisense	ACGUGACACGUUCGGAGAATT

## Data Availability

The datasets generated and analyzed during this study are available from the corresponding author on reasonable request. All data and materials, as well as software applications, support their published claims and comply with field standards.

## Abbreviations

The following abbreviations are used in this manuscript:

CMT	Canine mammary tumor
CCK-8	Cell Counting Kit-8
COX-2	Cyclooxygenase-2
CTCs	Circulating tumor cells
GSK-3	Glycogen synthase kinase-3
HCC	Hepatocellular carcinoma
HDACI	Histone deacetylase inhibitor
H&E	Hematoxylin and Eosin
lncRNAs	Long non-coding RNAs
MRCC	Mitochondrial respiratory chain complexes
PGE2	Prostaglandin E2
ROS	Reactive oxygen species
RT-qPCR	Real-time quantitative PCR
